# The role of class IIa histone deacetylases in regulating endothelial function

**DOI:** 10.3389/fphys.2023.1091794

**Published:** 2023-03-01

**Authors:** Zexu Shen, Yun Bei, Haoran Lin, Taofeng Wei, Yunjian Dai, Yangmin Hu, Chao Zhang, Haibin Dai

**Affiliations:** ^1^ Department of Pharmacy, The Second Affiliated Hospital of Zhejiang University School of Medicine, Hangzhou, China; ^2^ Department of Pharmacy, The First People’s Hospital of Hangzhou Lin’an District, Hangzhou, China

**Keywords:** histone deacetylases, endothelial cells, angiogenesis, atherosclerosis, inflammation, inhibitor

## Abstract

Vascular endothelial cells (ECs) are monolayer cells located in the inner layer of the blood vessel. Endothelial function is crucial in maintaining local and systemic homeostasis and is precisely regulated by sophisticated signaling pathways and epigenetic regulation. Endothelial dysfunctions are the main factors for the pathophysiological process of cardiovascular and cerebrovascular diseases like atherosclerosis, hypertension, and stroke. In these pathologic processes, histone deacetylases (HDACs) involve in epigenetic regulation by removing acetyl groups from lysine residues of histones and regulating downstream gene expression. Among all HDACs, Class IIa HDACs (HDAC4, 5, 7, 9) contain only an N-terminal regulatory domain, exert limited HDAC activity, and present tissue-specific gene regulation. Here, we discuss and summarize the current understanding of this distinct subfamily of HDACs in endothelial cell functions (such as angiogenesis and immune response) with their molecular underpinnings. Furthermore, we also present new thoughts for further investigation of HDAC inhibitors as a potential treatment in several vascular diseases.

## 1 Introduction

The endothelium is constituted by a thin layer of endothelial cells covering the inner lumens of blood vessels and lymphatic vessels, which regulate regional blood flow and fluid exchange. In addition, endothelium regulates vessel permeability and immune infiltration across the vascular wall, and synthesizes and secretes various biologically active substances (Nadar et al., 2004). Therefore, maintaining normal endothelial function is crucial for vascular homeostasis. Endothelial dysfunction and vascular homeostasis deficiency are the main features of vascular diseases (Butt et al., 2010). There is increasing evidence that endothelial dysfunction has a close relationship to the progression of coronary artery disease (CAD), atherosclerosis, and chronic kidney disease (CKD) (Vanhoutte, 1997; Landmesser et al., 2004; Zoccali et al., 2017). The molecular and cellular underpinnings in endothelial function and their role in vascular disease pathogenesis, diagnosis, treatment, and prognosis remain unknown.

Epigenetic modification is essential in the reversible regulation of gene expression with no alteration to the DNA sequence (Pons et al., 2009). DNA and histone tail modification are two primary means of epigenetic processes (Bonasio et al., 2010). Histone acetyltransferases (HATs) and histone deacetylases (HDACs) regulate transcription through acetylation and deacetylation of histones, leading to the relaxation and condensing of nucleosomes respectively (Verdin and Ott, 2015). These alterations in chromosome structure and DNA-binding affinity regulate the expression of genes (Sadoul et al., 2011). HDACs are critical transcriptional cofactors that are recruited by other sequence-specific transcription factors and play essential roles in vascular homeostasis and vessel development (Zhao et al., 2020). Among all HDACs, class I HDACs (HDAC1, HDAC2, HDAC3, and HDAC8) and class IIa HDACs (HDAC4, HDAC5, HDAC7, and HDAC9) are the most well-studied because of their close relationship to vascular biology and pathology. Improving the understanding of class IIa HDACs in endothelial functions will be conductive to develop new therapeutic strategies for vascular diseases.

In this review, we focus on the functions of class IIa HDACs in the endothelium and summarize the currently discovered mechanisms, molecular and cellular underpinnings. We attempt to clarify the connection between several particular cardiovascular diseases and their probable molecular mechanism from the perspective of endothelial function. Moreover, we also discuss the potential therapeutic effect of class IIa HDAC inhibitors (HDACi) in several specific vascular indications.

## 2 Class IIa HDACs

**FIGURE 1:**
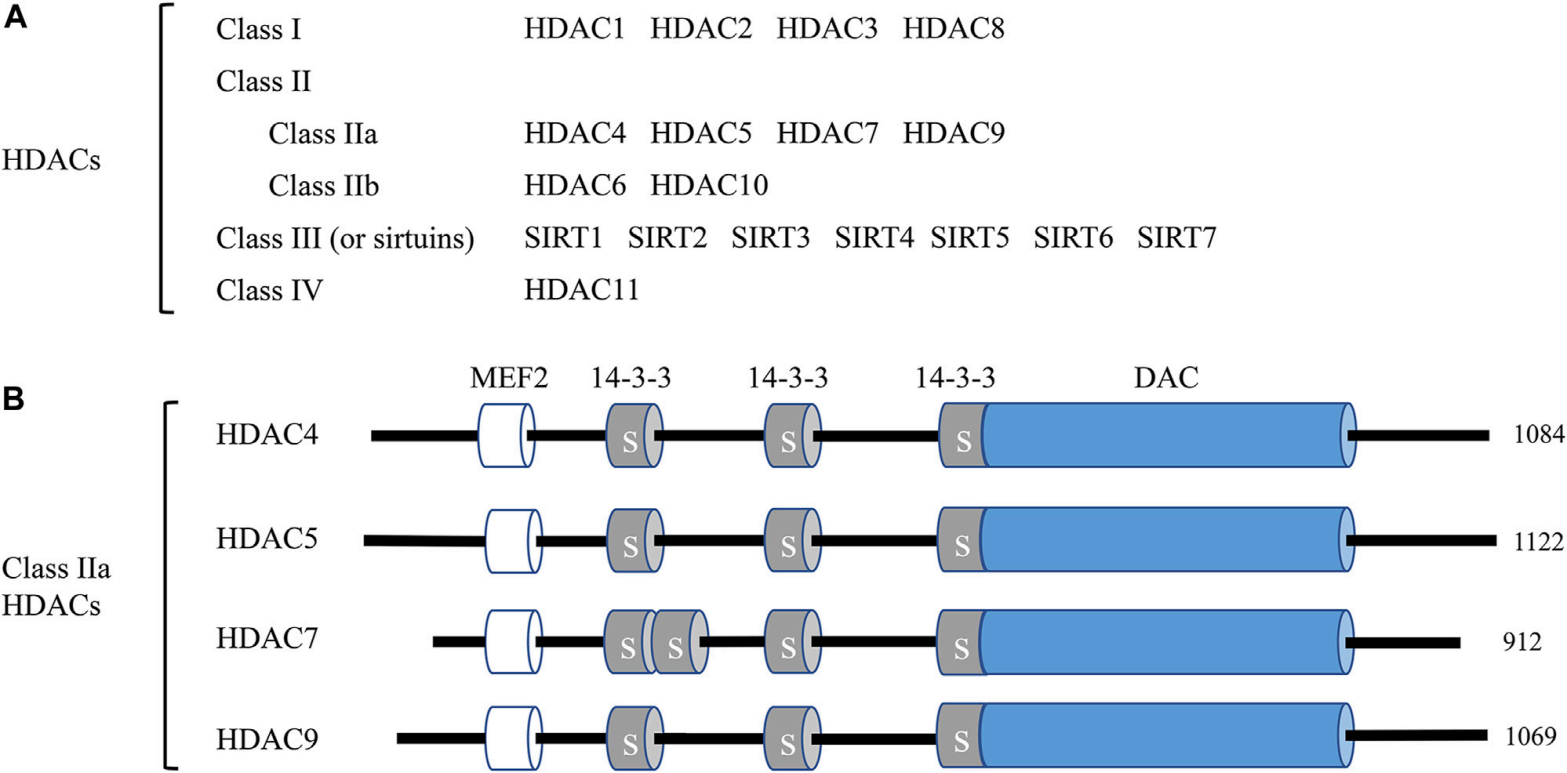
Classification of histone deacetylases (HDACs) and the domain organization of class IIa HDACs. **(A)** A total of 18 mammalian HDACs are categorized into four classes. Including HDAC11, all HDACs are involved in endothelial cell function at different levels and by different mechanisms. **(B)** The domain organization of class IIa HDACs. Class IIa HDAC members HDAC4, HDAC5, HDAC7, and HDAC9 and their total amino acid lengths are shown on the right. MEF2, myocyte enhancer factor 2 (MEF2) binding domain; 14-3-3, 14-3-3 binding motifs, label S is for Ser; DAC, deacetylation domain.

HDACs are enzymes that remove acetyl functional groups of the ε-N-acetyl-lysine amino acid from histone and non-histone proteins, which controls the epigenetic regulation of gene expression in multiple cellular processes (Ali et al., 2018). Currently, researchers have identified 18 mammalian HDACs, and they are divided into four subclasses based on their sequence and their active mechanism: class I members, HDAC1, 2, 3, and 8; class II members, HDAC4, 5, 6, 7, 9, and 10 (further divided into two subtypes, IIa: HDAC4, 5, 7, 9 and IIb: HDAC6 and 10); class III members, SIRT1-7; and the class IV member, HDAC11 (Seto and Yoshida, 2014). The complete list of HDACs and their classification are presented in Figure 1A. The class I and II HDAC proteins are zinc-dependent hydrolases, while the proteins of class III HDACs are NAD^+^-dependent (Witt et al., 2009). Various studies imply that HDACs are correlated with the pathological process of numerous human diseases, including cancer (Boggs et al., 2015), cardiovascular disease (CVD) (Xie and Hill, 2013), and neurological disorders (Govindarajan et al., 2013).

Class IIa HDACs (HDAC 4, 5, 7, and 9) exert unique characteristics from other HDACs. All class IIa HDACs embody the characteristics of a C-terminal deacetylase domain (DAC), a nuclear export sequence (NES), and an N-terminal adapter domain containing a myocyte enhancer factor 2 (MEF2) and a 14-3-3 domain. The schematic diagram of class IIa functional domains is shown in Figure 1B. The MEF2 binding domains are critical for the nuclear localization of class IIa HDACs, while the 14-3-3 domains are critical for the cytoplasmic localization through the phosphorylation of Ser residues (Yang and Gregoire, 2005). Compared to class I HDACs, class IIa HDACs exhibit a much more limited enzymatic activity under specific conditions and act as signal transducers shuttling between the nucleus and cytoplasm of cells (Martin et al., 2007). Researchers have identified several roles of class IIa HDACs in endothelial homeostasis. For example, Chang S et al. reported that HDAC7 gene disruption caused endothelial adhesion failure, loss of vessel integrity, and embryonic lethality (Chang et al., 2006).

## 3 Angiogenesis

Angiogenesis is a normal and essential process for human physiological and pathophysiological processes, including organ development, wound healing, and the formation of granulation tissue. However, it is also a fundamental step in the transformation of aneurysms from a benign to a malignant state, which is why angiogenesis inhibitors are used in cancer therapy (Milosevic et al., 2022). Angiogenesis also plays a critical role in the pathogenesis of tumor growth, atherosclerosis, diabetic retinopathy, and several inflammatory autoimmune diseases such as rheumatoid arthritis (RA), spondyloarthropathies, psoriasis, systemic lupus erythematosus, systemic sclerosis, and atherosclerosis (Carmeliet and Jain, 2000; Elshabrawy et al., 2015). Physiologically, it can be activated by mechanical stimulation and/or chemical stimulation. The vascular endothelial growth factor (VEGF) family and its receptor, vascular endothelial growth factor receptor (VEGFR), are crucial for the angiogenesis process and endothelial cell (EC) function (Ferrara et al., 2003; Ha et al., 2008a; Carmeliet and Jain, 2011). In this section, we review how class IIa HDACs affect endothelial angiogenesis by regulating gene transcription.

### 3.1 Role of HDAC4 in endothelial angiogenesis

**FIGURE 2:**
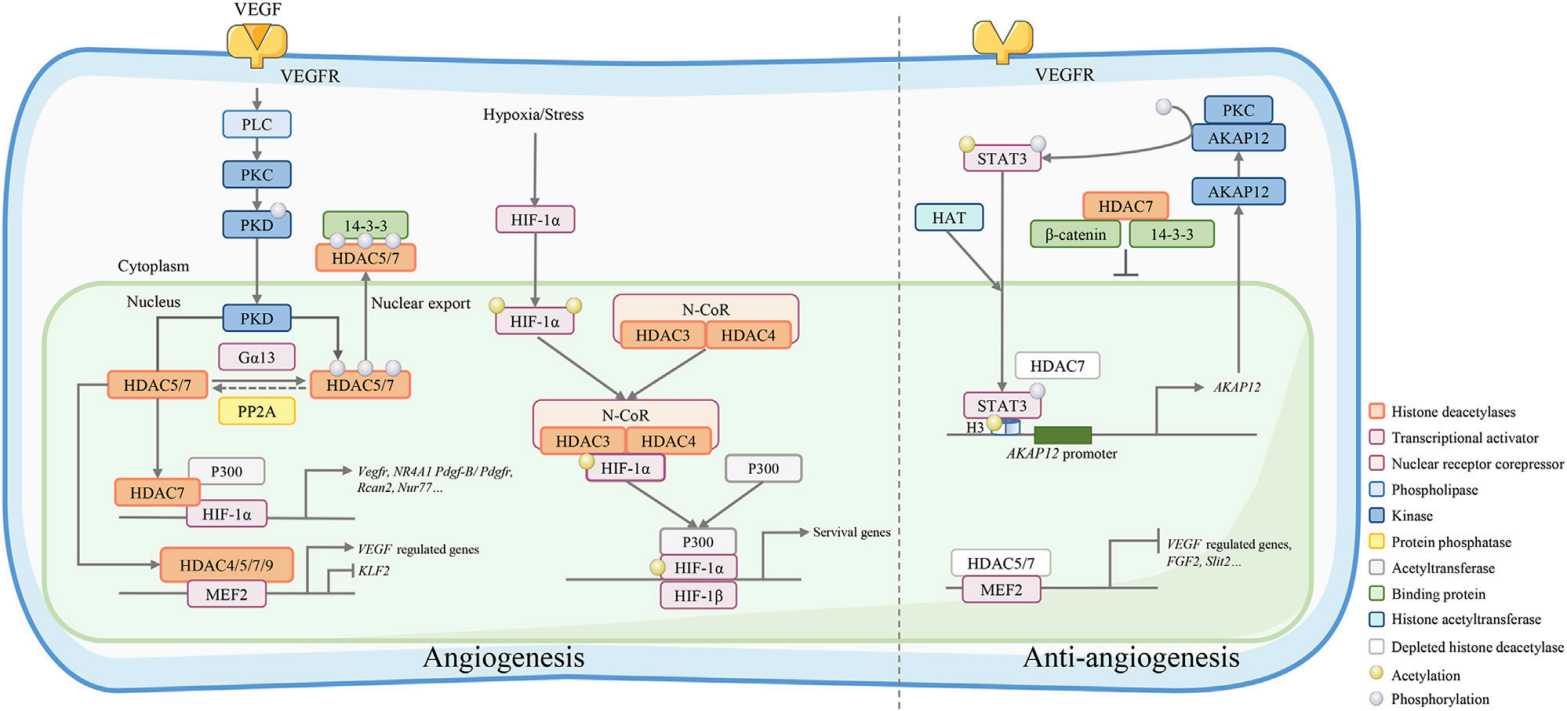
Class IIa HDACs play an important role in angiogenesis regulation. VEGF binds to VEGFR and activates its downstream phospholipase C (PLC)-protein kinase C (PKC)-protein kinase D (PKD) signaling. The activated kinase PKD phosphorylates HDAC7 to activate 14-3-3 binding sites and resulting in the export of HDAC7 to the cytoplasm. Conversely, phosphorylated IIa HDACs can be dephosphorylated by protein phosphatase 2A (PP2A) and regulate gene expression by forming a complex with HIF-1α and acetyltransferase p300 or a complex with MEF2. Under hypoxic/stressful conditions, the HDAC4/N-CoR/HDAC3 complex binds to hypoxia-inducible factor (HIF)-1α and represses basal transcription in endothelial cells. HDAC7 depletion in endothelial cells leads to repressed migration and tube formation with overexpression of A-kinase anchor protein 12 (AKAP12). AKAP12 interacts with PKC and phosphorylates transducer and activator of transcription 3 (STAT3). Phosphorylated STAT3 increasingly binds to H3 histones associated with the AKAP12 promoter, thus increasing the mRNA and protein levels of AKAP12. In the cytoplasm, HDAC7 can also bind the 14-3-3 protein and β-catenin to stabilize β-catenin, which stagnates HDAC7 in the nucleus and inhibits endothelial cell growth.

HDAC4 phosphorylation is essential for angiogenesis after cerebral ischemia. In a primary rat brain microvascular endothelial cell (RBMEC) hypoxia model, blockage of HDAC4 phosphorylation significantly decreased downstream hypoxia-inducible factor (HIF)-VEGF signaling (Liu et al., 2017). HDAC4 phosphorylation enhances EC motility in wound healing assays and facilitates EC tube formation. In the research of tasquinimod, an orally active anti-angiogenic drug, John Isaacs et al. considered HDAC4 as the target of its anti-angiogenic activity. Their study revealed that tasquinimod allosterically binds to HDAC4 at the regulatory Zn^2+^ binding domain and prevents HDAC4/nuclear receptor corepressor (N-CoR)/HDAC3 complex formation, which inhibited HDAC4-regulated histone deacetylation and transcription, such as HIF-1α, a transcription factor binding to promoters/enhancers (Isaacs et al., 2013). Thus, HDAC4 is necessary for epigenetic reprogramming in cell survival and angiogenesis responses (Figure 2).

H_2_O_2_ can oxidize HDAC4 and increase its phosphorylation. Disruption of the HDAC4/MEF2A complex is caused by NADPH oxidase 4 (Nox4)-derived H_2_O_2_, thereby inhibiting MEF2A activity and eventually ensuring normal EC tubular formation (Miska et al., 1999; Schader et al., 2020). In human umbilical vein endothelial cells (HUVECs) and human mammary epithelial cells (HMECs), HDAC4 overexpression results in impaired tube formation, whereas redox-insensitive HDAC4 has no exhibition effect. Nox4 expression is induced by H_2_O_2_ and transforming growth factor beta (TGFβ) treatment of cells, and Nox4 co-overexpression can induce phosphorylation of HDAC4 and restore the inhibition of tube formation by HDAC4 (Helfinger et al., 2016). In summary, Nox4 oxidizes HDAC4, increases its phosphorylation, and ultimately ensures proper tube formation in endothelial cells (Schader et al., 2020).

In the context of pulmonary arterial hypertension (PAH), MEF2 in pulmonary artery endothelial cells (PAECs) significantly decreases the expression of pulmonary vascular homeostasis-related genes, such as microRNAs 424 and 503, connexins 37 and 40, and kruppel-like factors (KLF) (Kim et al., 2013). The excessive nuclear accumulation of HDAC4 and HDAC5 impairs MEF2 activity in PAH PAECs, inhibiting their functions and reducing cell migration and proliferation, thus alleviating the injury of experimental pulmonary hypertension models (Kim et al., 2015).

VEGFR2 activation is the most downstream signaling pathway mediated by VEGF. HDAC4 and 5 are preferentially involved in VEGFR-2 expression (Hrgovic et al., 2017). The dynamic control of two deacetylases, HDAC5 and HDAC6, and the acetyltransferase p300 regulates VEGFR2 acetylation in endothelial cells and directly affects VEGFR2 function (Zecchin et al., 2014a). Five reported acetylation sites for human VEGFR2 are Lys929, Lys937, Lys939, Lys947, and Lys1053 (Zecchin et al., 2014b). VEGF-induced VEGFR2 phosphorylation is significantly increased by acetylation and results in the activation of additional proteins that regulate lysine acetylase activity, including cyclin-dependent kinase 2 (Cdk2), Cdk9, and p38 (Pillai et al., 2011).

### 3.2 Role of HDAC5 in endothelial angiogenesis

HDAC5 is a suppressor of endothelial cell angiogenesis. Among other class IIa HDACs, HDAC5 silencing exhibits unique features that stimulate endothelial cell migration, sprouting, and tube formation (Urbich et al., 2009). In HUVECs, the HDAC5 phosphorylation and nuclear export are stimulated by VEGF *via* the VEGF receptor 2-phospholipase Cγ (PLC)- protein kinase C (PKC)-protein kinase D (PKD)-dependent pathway. The PKD-HDAC5 pathway mediates MEF2 transcriptional activation and specific gene expression as a response to VEGF, such as nuclear receptor subfamily 4 group A member 1 (NR4A1), an angiogenesis-related orphan nuclear receptor. HDAC5 mutants specifically lack PKD-dependent phosphorylation and inhibit VEGF-mediated NR4A1 expression, EC migration, and angiogenesis *in vitro* (Ha et al., 2008b). HDAC5 overexpression alleviates EC sprout formation (Ha et al., 2008b). The anti-angiogenesis role of HDAC5 is unrelated to its deacetylase activity and MEF2 binding. A microarray expression analysis of HUVECs showed that HDAC5 silencing increased the expression of the angiogenic guidance factors slit guidance ligand 2 (SLIT2) and fibroblast growth factor 2 (FGF2). Chromatin immunoprecipitation assays have also shown that HDAC5 binds to FGF2 and Slit2 promoters, although further investigations are required to address the specific underlying mechanism (Urbich et al., 2009).

The peptide ligand apelin and its receptor (APJ) and the α subunit of heterotrimeric G13 protein (Gα13) play essential roles in vascular development (Offermanns et al., 1997). *Apj* ^*−/−*^ mice exhibit severe cardiovascular developmental defects that can cause embryonic lethality. The underlying mechanism of this effect involves Gα13 inducing phosphorylation and cytoplasmic translocation of HDAC4 and HDAC5, activating MEF2 and affecting the expression of transcription target kruppel-like factor 2 (KLF2) (Kang et al., 2013). *APJ* knockout increases the nuclear accumulation of HDAC4 and HDAC5 in ECs. However, constitutively activated Gα13Q226L derepresses HDAC5-mediated inhibition of gene transcription and induces HDAC5 subcellular translocation (Liu et al., 2009). The repression of MEF2-dependent gene transcription follows the inhibition of HDCA5. Therefore, HDAC5 is an essential component in Gα13-mediated angiogenesis.

### 3.3 Role of HDAC7 in endothelial angiogenesis

Endothelial cell proliferation is a crucial step for angiogenesis. Overexpression of HDAC7 by adenovirus gene transfection inhibits HUVEC proliferation, inhibits β-catenin nuclear translocation, and downregulates T-cytokine-1/Id2 (DNA binding inhibitor 2) and cyclin D1, resulting in a prolonged G1 phase (van de Wetering et al., 2002). In this process, HDAC7 stabilizes β-catenin in the cytoplasm by acting as a bridge between 14-3-3 protein and β-catenin, thereby inhibiting EC growth. VEGF treatment increases the degradation of HDAC7, resulting in the release of β-catenin from the HDAC7-β-catenin-14-3-3 complex and translocation to the nucleus (Ha et al., 2008a). The above effect results in increased expression of the β-catenin target gene and regulation of the growth of endothelial cells (ECs).

HDAC7 and its target genes are involved in VEGF-stimulated EC proliferation, differentiation, migration, tube formation, and microvessel sprouting (Ha et al., 2008a). HDAC7 and other class IIa HDACs are phosphorylated on Ser178, 344, and 479 conservatively in a dose- and time-dependent manner *via* the PLC/PKC/PKD1-dependent signaling pathway.

The phosphorylated HDAC7 exports from the nucleus to the cytoplasm and activates VEGF-responsive genes, such as matrix metallopeptidase 10 (MMP10), and affects EC proliferation and migration (Figure 2) (Wang et al., 2008; Liu et al., 2016). Protein phosphatase 2A (PP2A), a phosphatase dephosphorylating the 14-3-3 binding sites of class IIa HDACs, is also an important regulator of EC angiogenesis. Dephosphorylation of HDAC7 by PP2A controls its subcellular localization and represses its activity, affecting EC angiogenesis (Martin et al., 2008). PKD1 inhibitor can partly inhibit the VEGF-A increased phosphorylation of HDAC7 and its nuclear export, but not by the PI3K or MEK inhibitor. HDAC7 silencing impairs the migration and tube formation of endothelial progenitor cells (EPCs) *via* the VEGF-PKD1-HDAC7 axis (Yu et al., 2014), thus inhibiting VEGF-induced angiogenic gene expression, such as MMP10 (Ha et al., 2008a; Wang et al., 2008). Mottet, D et al. also demonstrated that HDAC7 silencing inhibited EC migration with no effects on apoptosis or adhesion of ECs. HDAC7 silencing inhibits tube formation *in vitro* and increases the expression of platelet-derived growth factor (PDGF) and its receptor PDGFR-β. Unlike HDAC4 or HDAC5, the exportation of HDAC7 out of the nucleus is associated with the PKC/PKD pathway instead of the Ca^2+^/camodulin-dependent kinase pathway (Mottet et al., 2007).

HDAC7 also epigenetically regulates AKAP12. Removal of HDAC7 triggers PKC-dependent phosphorylation and signal transducer and activator of transcription 3 (STAT3) acetylation. By recruiting histone acetyltransferase, the activated STAT3 acetylates the histone H3 on the AKAP12 gene promoter, resulting in elevated AKAP12 expression, which negatively regulates HUVEC migration and inhibits the formation of tubular structures (Figure 2) (Turtoi et al., 2012). Neuron-derived clone 77 (Nur77) and regulator of calcineurin-2 (RCAN2) are two essential genes related to angiogenesis (Wang et al., 2008). The expression levels of Nur77 and RCAN2 are increased in MCAO rats, and this can be reversed by suppressing HDAC4 phosphorylation.

MicroRNA143 (MiR-143) is expressed in both osteoblast cells and a specific subtype of CD31^hi^endomucin^hi^ endothelial cells, which have been shown to promote osteoblast development and bone formation. MiR-143 directly targets the inhibitory factor HDAC7, and HDAC7 knockdown is found to rescue the function of miR-143 deficiency (Wang et al., 2020). In addition, HDAC7 silencing in EPCs impairs the formation of vascular function and promotes tumor progression by inhibiting angiogenesis through transcriptional regulation of proangiogenic and antiangiogenic genes. These results suggest that HDAC7 is a promising target for antiangiogenic therapy in EPCs (Wei et al., 2018).

### 3.4 Role of HDAC9 in endothelial angiogenesis

Both *in vivo* and *in vitro* experiments illustrate that HDAC9 exerts significant pro-angiogenic effects (Kaluza et al., 2013). Similar to other class IIa HDACs, the nuclear location of HDAC9 is the basis for its biological activity. HDAC9 ^−/−^ mice present reduced postnatal retinal vessel formation and blood flow recovery in the hindlimb ischemia model. MicroRNAs are endogenous small non-coding RNAs that inhibit the translation process to initiate mRNA degradation and play an essential role in epigenetic regulation (Guo et al., 2010). The miR-17–92 cluster is a conservative negative regulator of angiogenesis in endothelial cells (Doebele et al., 2010). Interestingly, the role of HDAC9 in angiogenesis is closely correlated with the miR-17–92 cluster. HDAC9 silencing and miR-17–92 cluster blocking have an antiangiogenic effect.

## 4 Inflammatory signaling

**FIGURE 3:**
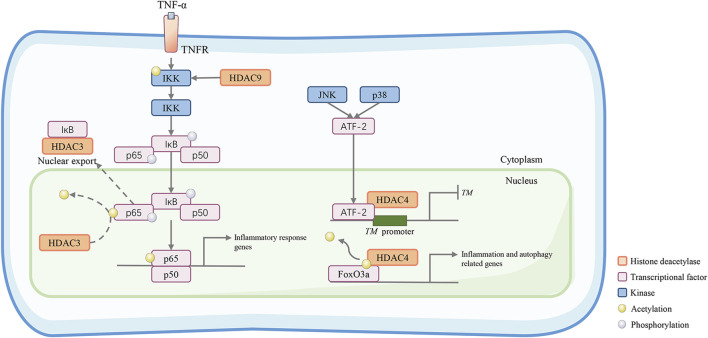
Class IIa HDACs promote inflammatory and atherosclerotic disease progression. HDAC9 activates the inhibitory kappa B kinase/nuclear factor-kappa B (IKK/NF-κB) pathway to induce vascular inflammation. HDAC9 reduces IKK acetylation. This results in reduced IKK activity, increased p65 phosphorylation, and increased proinflammatory responses in HUVECs. HDAC3 can deacetylate the NF‐κB subunit p65, which promotes its nuclear export. In the C-Jun N-terminal kinase (JNK) and p38 pathways, ATF-2 can recruit HDAC4 and bind to the TM promoter to form a transcriptional repression complex, which may diminish the gene transcription of the TM gene, an important vascular protective molecule in anti-inflammation. In addition, HDAC4 deacetylates the transcription factor forkhead box O3a (FoxO3a) allowing it to act to increase the transcriptional activity of autophagy- and inflammation-related genes.

Vessel inflammation is the cause of vascular blockage, ischemia, and necrosis, which are associated with the development and progression of vascular calcification, diabetic vascular dysfunction, and atherosclerosis (Pober and Sessa, 2007). It has been reported that angiotensin II (Ang II) is involved in vascular inflammation by the overexpression of vascular adhesion molecule-1 (VCAM-1) and inducible nitric oxide synthase (iNOS), cyclooxygenase-2 (COX-2), and interleukin-6 (IL-6) (Levick et al., 2010). HDAC4 mediates Ang II-induced vascular inflammation. Data show that HDAC4 is rapidly upregulated in vascular endothelial cells with increased autophagic flux and inflammatory mediators. HDAC4-mediated expression of forkhead box O3a (FoxO3a) is the most likely mechanism of the above effects of HDAC4. Moreover, the upregulation of HDAC4 leads to the deacetylation of autophagy-related genes and activation of the FoxO3a transcription factor, subsequently causing vascular inflammation (Figure 3) (Yang et al., 2018). HDAC4 knockdown or FoxO3a silencing significantly ameliorated vascular inflammation caused by Ang II.

KLFs are a subfamily of the zinc-finger class of DNA-binding transcription factors, which are potent and critical regulators of endothelial proinflammatory activation (Feinberg et al., 2004). In HUVECs, tumor necrosis factor-alpha (TNF-α) represses the expression of KLF2 *via* the nuclear factor-kappa B (NF-κB) pathway. However, a constitutively active form of IκBα or treatment with the HDAC inhibitor trichostatin A dramatically abrogates this effect, which signifies that HDACs may be involved in this process. Chromatin immunoprecipitation assays and coimmunoprecipitation assays demonstrated that HDAC4 and p65 synergistically inhibit the ability of MEF2 to induce the KLF2 promoter. The trimolecular complex, a form of p65, MEF2, and HDAC4, is crucial for the inflammatory response of vascular endothelial cells (Kumar et al., 2005). Furthermore, HDAC4 is also responsible for endothelial cell apoptosis. MiR-200b-3p is overexpressed after ECs receive stimuli from molecules such as oxidized low-density lipoprotein (ox-LDL) and reactive oxygen species (ROS), which inhibits the nuclear translocation of HDAC4, thereby repressing the expression of several apoptosis-related genes, e.g., B cell lymphoma 2 (BCL2) (Zhang et al., 2021).

The NF-κB family is composed of vital transcription factors contributing to inflammation and innate immunity. NF‐κB is bound to the inhibitory protein inhibitor of κB (IκB) and generally resides in the cytoplasm in its inactive form. In response to stimuli, IκB is phosphorylated by IκB kinases (IKKs) and degraded, which allows NF‐κB to translocate to the nucleus and exert biological activity (Oeckinghaus et al., 2011). Class IIa HDACs negatively regulate the biological function of NF‐κB by binding to IκBα (Munro et al., 2021). Moreover, the NF‐κB subunit p65 can be deacetylated by HDAC3, which promotes its binding to IkBα and its nuclear translocation. In HUVECs, HDAC9 knockdown reduces p65 phosphorylation at serine 536 and 468, which is mediated by cAMP-dependent protein kinase A catalytic subunit (PKAc), mitogen- and stress-activated protein kinase 1 (MSK1) and Moloney murine leukemia provirus integration site (PIM1) (Hoesel and Schmid, 2013). At the animal level, HDAC9 binds to the NF-κB activating kinases IKK-α and IKK-β, resulting in their deacetylation and subsequent activation, thereby motivating inflammatory responses in ECs, which act as a formative factor of atherosclerotic plaque vulnerability (Figure 3) (Asare et al., 2020). Interestingly, TMP195, a class IIa HDAC inhibitor, can diminish the activity of HDAC9 and suppress its binding to IKK-β, therefore alleviating vascular inflammation.

Ox-LDL-induced endothelial cell apoptosis and inflammation are crucial periods in the progression of atherosclerosis and can be regulated by HDAC9. In HUVECs, HDAC9 knockdown effectively decreases the expression of inflammatory factors, including TNF-α and monocyte chemoattractant protein 1 (MCP1) (Han et al., 2016). In addition, by regulating the circ_0003204/miR-942-5p/HDAC9 axis, HDAC9 acts downstream of circ_0003204 to influence ox-LDL-induced vascular inflammation (Wan et al., 2021).

## 5 Endothelial permeability

**FIGURE 4:**
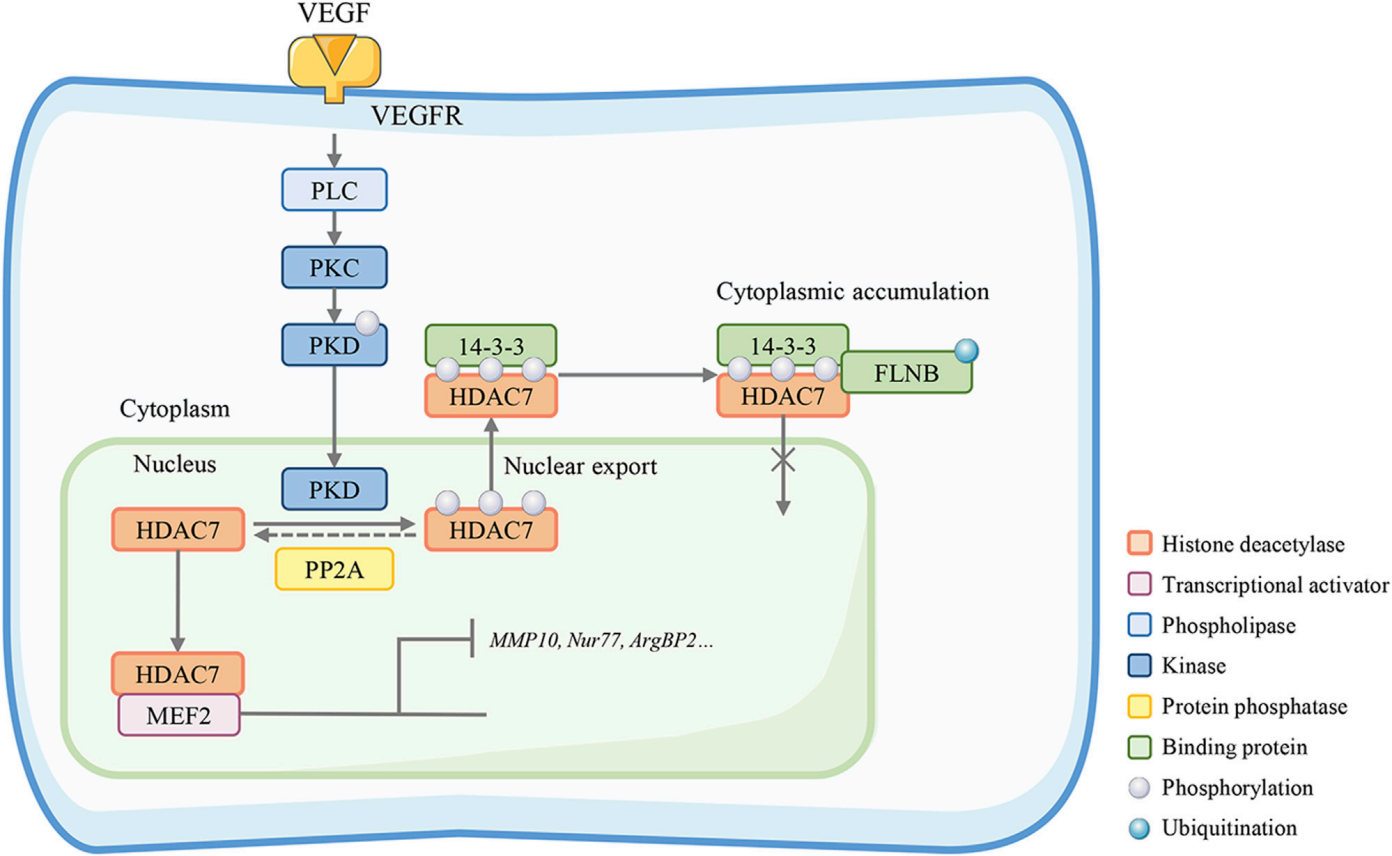
HDAC7 maintains endothelial permeability. Filamin B (FLNB)-mediated HDAC7 cytoplasmic sequestration is required in the VEGF-mediated spatiotemporal regulation, interacting with HDAC7 nuclear localization sequence and FLNB ubiquitination. The HDAC7 target genes MMP-10 and Nur77 are suppressed in this process. Knockout of FLNB or absent PP2A restores the HDAC7 nuclear-cytoplasmic trafficking and reduces HDAC7 target gene expression, especially MMP10, Nur77, and ArgBP2, thereby inhibiting vascular permeability.

HDAC7 activity can be regulated by the Bα regulatory subunit of PP2A (PP2A-Bα). PP2A-Bα silencing abrogates the repression of HDAC7 transcription, which leads to a cytoskeleton adaptor protein (ArgBP2) overexpression and inadequate rearrangements of the EC actomyosin cytoskeleton (Taieb et al., 2008). The PP2A-Bα/HDAC7/ArgBP2 axis plays a vital role in balancing endothelial cytoskeletal dynamics and cell-matrix adhesion to maintain the vascular lumens (Martin et al., 2013). Filamin B (FLNB) is a protein required for the regulation of VEGF-mediated temporal and spatial cytoplasmic separation of HDAC7. FLNB interacts with HDAC7 and this interaction involves the ubiquitination of FLNB and the nuclear localization sequence of HDAC7 (Xu et al., 2017). In human primary endothelial cells (HUVECs), small interfering RNA (siRNA) knockout of FLNB or ubiquitin (Ub) can block HDAC7 cytoplasmic accumulation mediated by VEGF and reduce HDAC7 target genes expression, especially the MMP10 and Nur77, thereby inhibiting vascular permeability induced by VEGF. (Figure 4) (Su et al., 2013). MMP10 is a secretory terminal protease that degrades the extracellular matrix and disrupts endothelial cell-cell adhesion during early embryogenesis. HDAC7 inhibits MMP10 gene transcription by binding MEF2, a direct activator of MMP10 transcription and an essential regulator of vascular development (Chang et al., 2006). In mice with HDAC7 gene disruption, MMP10 is overexpressed, resulting in consequent dilatation, rupture of blood vessels, and embryonic lethality.

## 6 Nitric oxide-induced endothelial functions

Nitric oxide (NO) is produced by endothelial nitric oxide synthase (eNOS) and plays a crucial role in modulating endothelial cell proliferation, inflammation, and redox homeostasis (Giles et al., 2012). HDAC5 regulates the flow-induced production of eNOS in HUVECs. When cells receive laminar shear stress (SS), AMP-activated protein kinase (AMPK) and calmodulin-dependent protein kinase IIα (CamK IIα) are activated and promote HDAC5 phosphorylation. Ser 259 and 498 phosphorylation sites are beneficial for HDAC5 nuclear export (Park et al., 2021). The dissociation of HDAC5 and MEF2 induced by laminar blood flow dramatically enhances MEF2 transcriptional activity, which heightens the expression of KLF2 and its downstream genes (e.g., eNOS) (Wang et al., 2010). Another study in HUVECs also obtained similar results: HDAC5 silencing enhances KLF2 transcription and facilitates eNOS expression (Kwon et al., 2014). In addition, SS-induced NO production causes histone deacetylation and facilitates the nuclear shuttling of HDAC4 and HDAC5. The subcellular location of the above HDACs is modulated by PP2A. Using HDACi (MC1568 and MS27-27), Illi B *et al.* demonstrated that class II HDAC nuclear translocation relies on NO-dependent PP2A activation, thus affecting histone acetylation levels *in vitro* (Illi et al., 2008). Taken together, class IIa HDACs and their translocation in the nucleus and cytoplasm regulate NO production and control endothelial functions.

## 7 Endothelial-mesenchymal transition

Under certain circumstances, ECs lose their original phenotype and turn to a myofibroblast-like phenotype, which is exhibited by decreased expression of endothelial markers (such as CD31 and VE-cadherin) and increased expression of mesenchymal markers (i.e., alpha-smooth muscle actin (α-SMA) and N-cadherin) (Souilhol et al., 2018). Endothelial-mesenchymal transition (EndMT) participates in the formation of cardiac valves in embryos and tissue rebuilding in adulthood (Kovacic et al., 2012; Kovacic et al., 2019). Interestingly, HDAC9 contributes to the EndMT process and promotes the progression of atherosclerosis. In HUVECs, overexpression of HDAC9 results in an increase in EndMT-related gene and protein expression, while mouse primary lung endothelial cells (MPLECs) extracted from endothelial-specific HDAC9 knockout (Endo-Hdac9KO) mice preserved a more endothelial-like phenotype. A study in an atherosclerosis animal model demonstrated that Endo-Hdac9KO or class IIa HDAC inhibitor (MC1568) treatment significantly attenuated the progression of atherosclerosis by reducing plaque area and enhancing plaque stabilization, even though further investigation is acquired (Lecce et al., 2021).

## 8 Coagulation process

Hemostasia and coagulation are crucial features of endothelial function. Thrombomodulin (TM), plasminogen activator inhibitor-1 (PAI-1), von Willebrand factor (vWF), and tissue plasminogen activator (t-PA) are primary molecules that maintain the balance of pro-coagulation and anticoagulation (Vallet and Wiel, 2001). Endothelial dysfunction disequilibrates the coagulation process and causes vascular thrombosis. Metabolic stress and inflammatory stimuli can activate the C-Jun N-terminal kinase (JNK) and p38 pathways and their target activating transcription factor-2 (ATF-2) (Vlahopoulos et al., 2008). In a fatty acid-induced model of human aortic endothelial cells, the expression of TM is inhibited. HDAC4 can be recruited when ATF-2 binds to the TM promoter, forming a transcriptional repression complex in the promoter, which may lead to chromatin condensation and transcriptional arrest, thereby regulating the coagulation process of endothelial cells (Rong et al., 2010) (Figure 3).

## 9 HADC inhibitors

HDAC inhibitors (HDACi) are chemicals that interfere with HDAC enzyme activity. In general, by inhibiting the function of HDAC enzymes, HDACis can increase the transcriptional activity of specific genes (Monneret, 2005). In terms of endothelial functions, the expression of several crucial angiogenesis-related genes, such as VEGF, HIF-1α, and tyrosine-protein kinase receptor-2 (Tie-2), is suppressed when cells are treated with HDACis (Mottet and Castronovo, 2010). Currently, scientists have made significant progress in HDACi research. Several HDACis, such as vorinostat, valproic acid, and sodium butyrate, are approved for the treatment of T cell lymphoma, multiple myeloma, and neurological disorders (Duvic et al., 2007; Nebbioso et al., 2012). Class IIa HDAC inhibitors also showed strong antitumor effects by altering the tumor microenvironment and enhancing cell death (Kikuchi et al., 2015; Guerriero et al., 2017). In this review, we focus on class IIa-related HDACis. The list of HDACis discussed in this review is shown in Table 1; the HDAC specificity and their role in endothelial functions and mechanisms are also included in the table.

**TABLE 1:** List of HDAC inhibitors discussed in this review.

HDAC inhibitor	HDAC specificity	Effect EC function	Mechanisms	References
Valproic acid	Class I, IIa	Thrombopoiesis	Increases histone acetylation at the t-PA promoter induces t-PA expression	Larsson et al. (2012)
		Angiogenesis	Inhibits VEGFR-2 protein expression	Hrgovic et al. (2017)
		Inflammation	Antagonizes the inflammatory damage of vascular tissue	Zhong et al. (2021)
Sodium butyrate	Class I, II	Angiogenesis	Inhibits VEGFR-2 protein expression	Hrgovic et al. (2017)
Trichostatin A	Class I, II, IV	Hypertension	Alleviates HDAC4-mediated vascular inflammatory responses	Usui et al. (2012)
		I/R injury	Prevents I/R injury-induced activation of gene programs that include cell death and vascular permeability	Granger et al. (2008)
		Angiogenesis	Inhibits VEGFR-2 protein expression	Hrgovic et al. (2017)
TMP 195	Class IIa	Atherosclerosis	Limits proinflammatory responses	Asare et al. (2020)
MC1568	Class IIa	NO homeostasis	Abolishes NO-induced formation of macromolecular complexes and regulates downstream gene expression	Illi et al. (2008)
Tasquinimod	HDAC4	Inflammation	Allosterically binds to HDAC4 and prevents HDAC4/nuclear receptor corepressor (N-CoR)/HDAC3 complex formation, which inhibites HDAC4-regulated histone deacetylation and transcription	Isaacs et al. (2013); Shen et al. (2022)
		Angiogenesis		

From the perspective of medicinal chemistry, improved target selectivity of HDAC inhibitors may lead to better safety, especially when inhibiting isoforms other than HDAC1-3 which may overwhelm the efficacy by the side effects of HDAC1-3 inhibition (Ho et al., 2020).

Specific inhibitors targeting class IIa HDACs have three main deficiencies: 1) The catalytic sites of proteins are highly similar to those of Class I HDACs, making selective targeting quite difficult to achieve. 2) The legitimacy of targeting the catalytic site of proteins which has little enzymatically active against acetylated lysines. In the active site in the C-terminal region of IIa HDACs, the key tyrosine residue is replaced by a histidine, resulting in low catalytic activity. More electrophilic trifluoroacetyllysine-containing peptide substrates have been employed for HDAC inhibitors *in vitro* assays against these isoforms. Thus, a lack of activity observed in enzyme assays against HDAC4, HDAC5, HDAC7, and HDAC9 is a misleading measure of selectivity, as a compound may still be capable of binding to the active sites and thereby abrogate their non-enzymatic role in protein scaffolding (Ho et al., 2020). Targeting Class IIa HDAC domains can be an indirect strategy to impact Class I HDACs. This class IIa HDACs-driven deacetylation may become a more selected transcriptional reset with less toxicity. 3) The low enzyme activity makes it difficult to find a suitable method to measure the inhibition of class IIa HDAC. Up to now, to test the potency and specificity of a class IIa HDACis, there is a double-check approach. The trifluoroacetyl-lysine can be employed to evaluate the compound as a class IIa-specific substrate, while “classical” substrates, such as acetylated lysines, can be used to exclude its inhibitory activity against other HDAC classes (Di Giorgio et al., 2015).

One potential strategy to design selective IIa HDACs inhibitors is based on the binding mode of IIa HDACs and these ligands. Comparison of these structures with the HDAC8 substrate–mimetic complex, two published structures of the catalytic domain complexed with a trifluoromethyl ketone and an analogous hydroxamic acid (Mehrling and Chen, 2016; Porter and Christianson, 2017) revealed the existence of a lower pocket in HDAC4 (Bertrand, 2010). For HDAC4 and HDAC7, U-shaped inhibitors were can dock in a pocket that should be formed to accommodate a phenyl ring pointed inward the surface around the histidine involved in the catalytic reaction (Roche and Bertrand, 2016).

As reported, the main structural type of selective class IIa HDAC inhibitors is trifluoromethyl ketones (Bürli et al., 2013). TMP269, a highly selective inhibitor, is the representative compound containing a trifluoromethyloxadiazole zinc binding group (Lobera et al., 2013). The X-ray structure of HDAC7 with a TMP269 ligand revealed an interaction between fluorine and oxadiazole oxygen with the zinc from HDAC7, which is a weak electrostatic interaction with zinc rather than direct coordination. In The active sites of IIa HDACs, the replacement of the tyrosine to the smaller histidine allows the bulky trifluoromethyloxadiazole to combine in this much roomy substrate binding channel, leading to high selectivity. Pharmacologically, TMP269 alleviates pulmonary arterial hypertension and cerebral ischemia/reperfusion injury in mouse models (Ho et al., 2020).

One more example, tasquinimod, a highly selective HDAC4 inhibitor, inhibits unilateral ureteral obstruction-induced renal tubular cell injury and apoptosis by reduced expression of neutrophilgelatinase–associated lipocalin, Bax, and inhibition of caspase-3 (Shen et al., 2022). The *K*_*d*_ binding with HDAC4 is 10–30 nM. Its homology modeling suggests that allosteric binding locks HDAC4 in an inactive conformation which is unable to interact with HDAC3 (Isaacs et al., 2013). In endothelial cells, tasquinimod induced reduced vascular inflammation the same as HDAC4 silencing, suggesting a functional consequence between HDAC4 and tasquinimod interaction (Yang et al., 2018; Ho et al., 2020).

## 10 Conclusion and future approaches

In the current review, we summarized the crucial role of class IIa HDACs (HDAC4, 5, 7, and 9) in EC functions, mainly related to angiogenesis, inflammation signaling, and endothelial permeability. Accumulating studies have clarified the mechanisms of translocation of class IIa HDACs between the nucleus and cytoplasm, and class IIa HDAC-mediated regulation of protein expression is critically involved in angiogenesis and other EC functions. However, the comprehensive mechanisms underlying class IIa HDAC regulation of EC functions are sophisticated and need to be further investigated. Growing evidence suggests that class IIa HDACs control EC function and participate in numerous human CVD diseases, especially stroke, and atherosclerosis. Hence, HDAC inhibitors (HDACis) may be a potential therapeutic strategy for treating relevant CVD diseases. Due to the similar structure and function of HDACs, scientists should pay more attention to the research and development of particular and selective HDAC inhibitors.

## Glossary

AKAP12: A-kinase anchoring protein 12
AMPK: AMP-activated protein kinase
Ang II: Angiotensin II
APJ: Apelin receptor
ArgBP2: Arg/c-Abl kinase binding protein 2
ATF-2: Activating transcription factor-2
BCL2: B cell lymphoma 2
CAD: Coronary artery disease
CamK IIα: Calmodulin-dependent protein kinase II
CaMKIV: Calcium/calmodulin-dependent protein kinase IV
Cdk2: Cyclin-dependent kinase 2
Cdk9: Cyclin-dependent kinase 9
CKD: Chronic kidney disease
COX-2: Cyclooxygenase-2
CVD: Cardiovascular diseases
DAC: Deacetylase domain
EC: Endothelial cell
EndMT: Endothelial-mesenchymal transition
EPCs: Endothelial progenitor cells
FGF2: Fibroblast growth factor 2
FLNB: Filamin B
FoxO3a: Forkhead box O3a
Gα13: α subunit of heterotrimeric G13 protein
HAT: Histone acetyltransferases
HDAC: Histone deacetylases
HDACi: HDAC inhibitor
HIF-1α: Hypoxia-inducible factor-1α
HMEC: Human mammary epithelial cell
HUVEC: Human umbilical vein endothelial cell
IKK: IκB kinases
IL-6: Interleukin-6
iNOS: Inducible nitric oxide synthase
JNK: C-Jun N-terminal kinase
KLF2: kruppel-like factor 2
MEF2: Myocyte enhancer factor 2
MiR-143: MicroRNA143
MMP10: Matrix metallopeptidase 10
MPLECs: mouse primary lung endothelial cells
MSK1: Mitogen- and stress-activated kinase-1
N-CoR: Nuclear receptor corepressor
NES: Nuclear export sequence
NF-κB: Nuclear factor-kappa B
NO: Nitric oxide
Nox4: NADPH oxidase 4
NR4A1: Nuclear receptor subfamily 4 group A member 1
Nur77: Neuron-derived clone 77
ox-LDL: Oxidized low-density lipoprotein
PAECs: Pulmonary artery endothelial cells
PAH: Pulmonary arterial hypertension
PAI-1: Plasminogen activator inhibitor-1
PDGF: Platelet-derived growth factor
PDGFR-β: Platelet-derived growth factor receptor-β
PIM1: Moloney murine leukemia provirus integration site
PKAc: cAMP-dependent protein kinase A catalytic subunit
PKC: Protein kinase C
PKD: Protein kinase D
PKD1: Protein kinase D1
PLC: Phospholipase C
PP2A: Protein phosphatase 2A
RBMEC: Rat brain microvascular endothelial cells
RCAN2: Regulator of calcineurin-2
ROS: Reactive oxygen species
siRNA: Small interfering RNA
SLIT2: Slit guidance ligand 2
STAT3: Signal transducer and activator of transcription 3
TGF-β: Transforming growth factor-β
Tie-2: Tyrosine-protein kinase receptor-2
TM: Thrombomodulin
TNF-α: Tumor necrosis factor-α
tPA: Tissue plasminogen activator
Ub: Ubiquitin
VCAM-1: Vascular adhesion molecule-1
VEGF: Vascular endothelial growth factor
VEGFR: Vascular endothelial growth factor receptor
VPCs: Vascular progenitor cells
vWF: von Willebrand factor.
